# Supplementary material for: TGFα Promotes Chemoresistance of Malignant Pleural Mesothelioma
Source: Cancers (Basel). 2020 Jun 6;12(6):1484. doi: 10.3390/cancers12061484 (PMC7352199; doi:10.3390/cancers12061484)
Supplement: Supplementary file 1 [file cancers-12-01484-s001.zip › cancers-818039 supplementary final/cancers-818039-supplementary final.docx]

Supplementary Materials

TGFα promotes chemoresistance of malignant pleural mesothelioma

Bernard Staumont, Majeed Jamakhani, Chrisostome Costa, Fabian Vandermeers, Sathya Neelature Sriramareddy, Gaëlle Redouté, Céline Mascaux, Philippe Delvenne, Pascale Hubert, Roghaiyeh Safari and Luc Willems

**
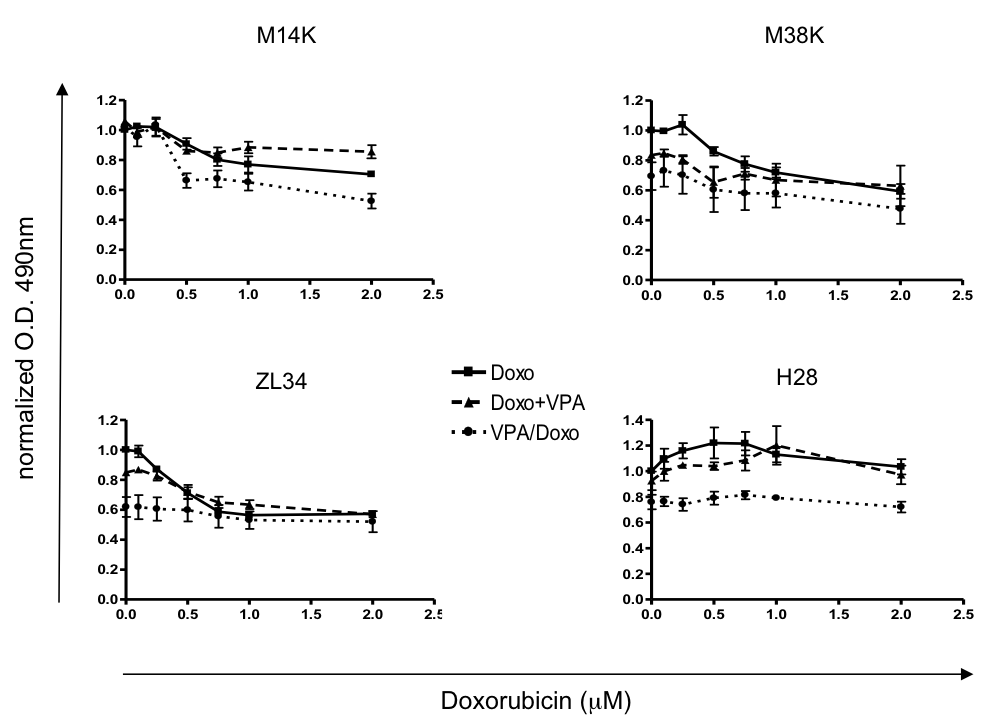
Figure S1.** Metabolic activity of MPM cell lines in the presence of doxorubicin and/or VPA. Four mesothelioma cell lines (M14K, M38K, ZL34 and H28; 10^4^ cells/mL in a 96-well plate) were incubated with different concentrations of doxorubicin (0–2 µM Doxo) alone or in combination with 2 mM of VPA added simultaneously (Doxo + VPA) or 24 hours earlier (VPA/Doxo). Cell viability was evaluated using the MTS assay (CellTiter 96 Aqueous One Solution Cell Proliferation assay, Promega). Twenty-four hours after adding doxorubicin, 20 µL of tetrazolium reagent was added to each well, and incubated for 2 h at 37 °C. Formazan precipitation was measured with a colorimetric microplate reader at a wavelength of 490 nm. Optical densities were normalized to mock condition arbitrarily set to 1.

**Figure S2.** Apoptosis of MPM cell lines in the presence of doxorubicin and/or VPA. Apoptosis was quantified using the annexin V-PE apoptosis detection kit (Becton Dickinson), which detects phosphatidylserine externalized in the early phases of apoptosis. Cells were plated at 10^5^ per mL in 24-well plates and treated with 2 mM of VPA and 0.5 µM of doxorubicin alone or in combination. After 48 hours, floating and adherent cells were collected, washed twice with cold PBS, resuspended in 100 µL of annexin binding buffer (10 mM Hepes, 140 mM NaCl, 2.5 mM CaCl_2_, pH 7.4), incubated for 15 min at room temperature with 5 µL of annexin V-PE and 5 µL of 7AAD and analyzed by flow cytometry (FACS Aria, Becton Dickinson). Ten thousand events were analyzed with the FACS Diva Software. * means *p* < 0.05 (Student’s t-test).

*

*

*

**Annexin V positive cells (%)**

**mock VPA doxo VPA+doxo**

**
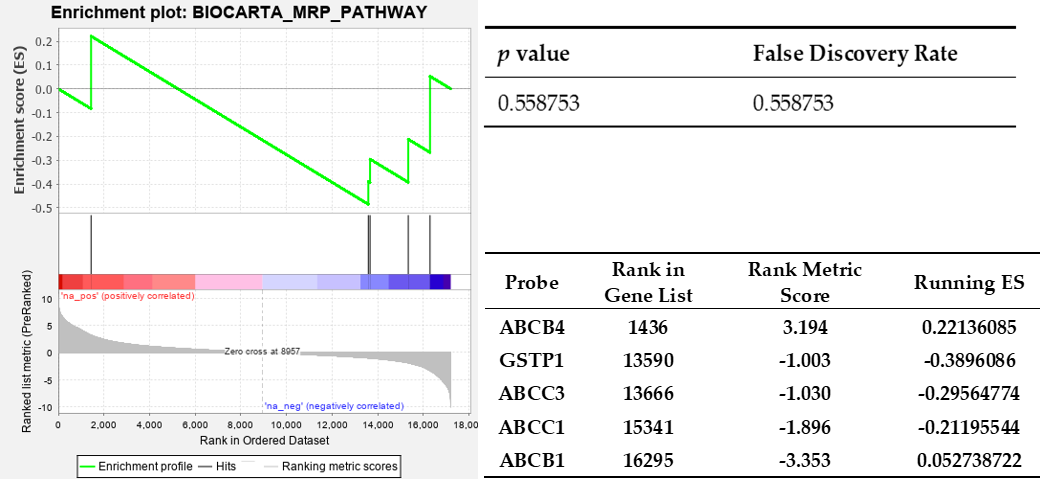
**

**Figure S3.** Enrichment plot of multidrug resistance pathway gene set using GSEA on the transcriptomic data of M14K and H28 cells. GSEA was performed with DESeq2 on the whole set of expressed genes ranked by their differential expression in M14K and H28 cell lines. The enrichment plot of the BIOCARTA MRP PATHWAY, the associated *p*-value and false discovery rate, as well as the list of genes involved, their rank and enrichment score are indicated.

**
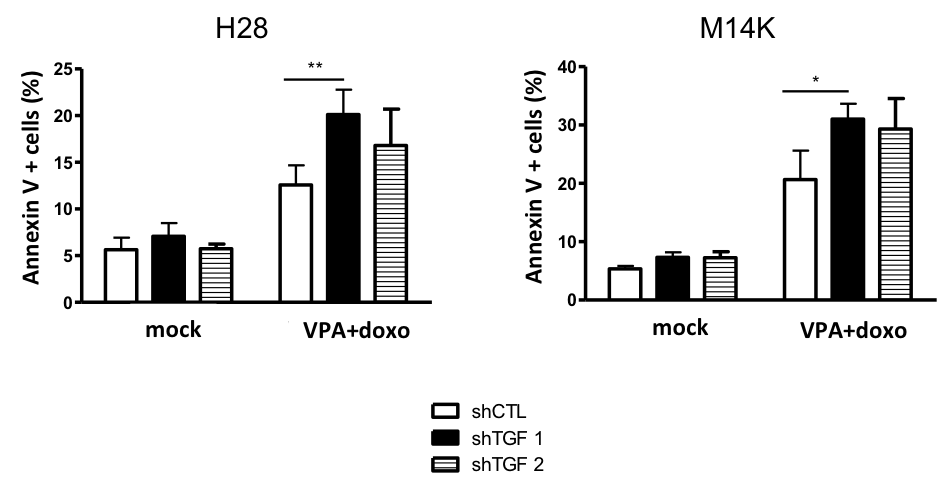
**

**Figure S4.** VPA + doxorubicin induced apoptosis upon transduction with a second TGFα shRNA. H28 and M14K cells were transduced with a lentiviral vector (pLKO.1-puro) expressing two different shRNAs directed against TGFα (shTGF 1 and shTGF 2). Apoptosis was quantified as described in Figure S2.


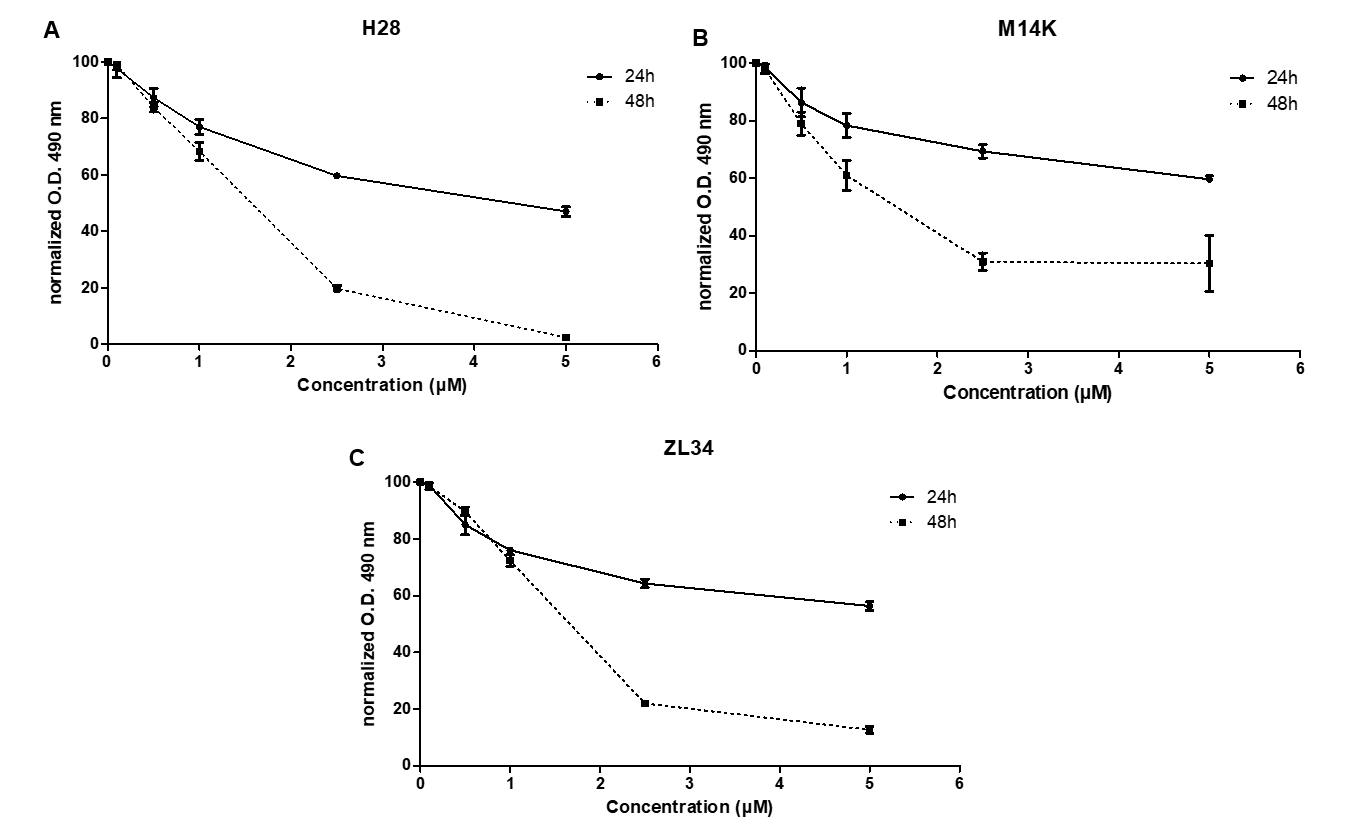


**Figure S5.** Metabolic activity of MPM cell lines in the presence of CUDC-101. **(A**) H28, **(B)** M14K, and **(C)** ZL34 mesothelioma cell lines (10^4^ cells/mL in a 96-well plate) were incubated with different concentrations of CUDC-101 inhibitor (0–5 µM). Cell viability was evaluated using the MTS assay (CellTiter 96 Aqueous One Solution Cell Proliferation assay, Promega). 24 or 48 h after adding CUDC-101, 20 µL of tetrazolium reagent was added to each well, and incubated for 2 h at 37 °C. Formazan precipitation was measured with a colorimetric microplate reader at a wavelength of 490 nm. Optical densities were normalized to mock condition arbitrarily set to 100.


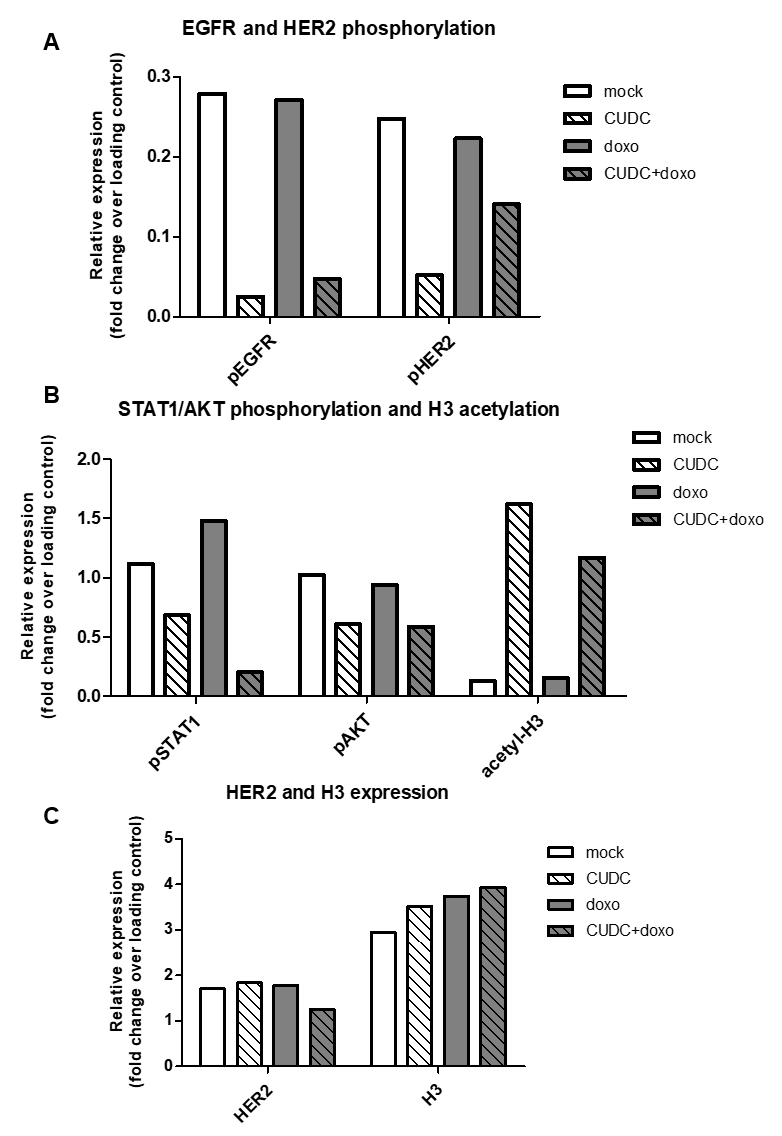


**Figure S6.** Immunoblotting intensity ratios. Each band of the immunoblotting of H28 cell lysates evaluating **(A)** the phosphorylation of EGFR and HER2; **(B)** the phosphorylation of STAT1 and AKT and H3 acetylation; **(C)** HER2 and H3 expression in response to doxorubicin and/or CUDC-101 was quantified relative to loading control (HSP90) using ImageJ software.


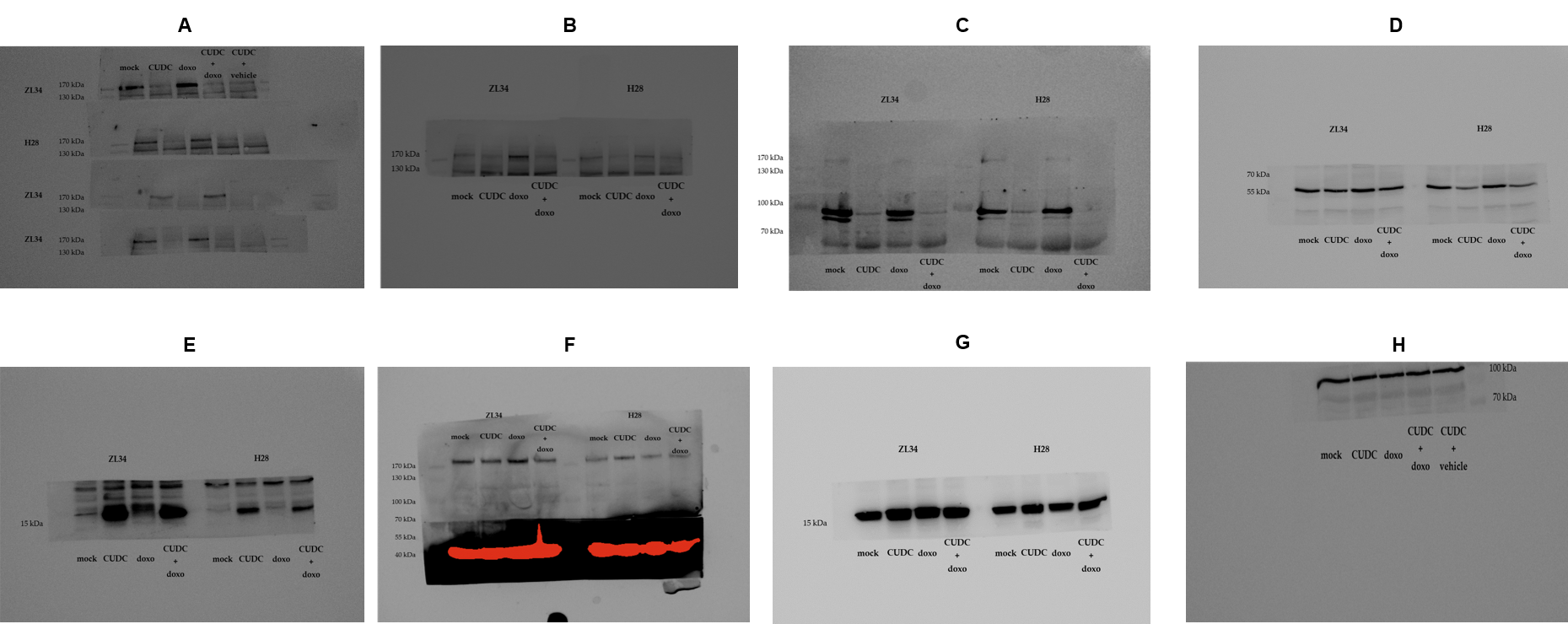


**Figure S7.** Original immunoblots. Original immunoblots of H28 and ZL34 cell lysates evaluating the phosphorylation of EGFR, HER2, STAT1, and AKT (panels A to D respectively), acetylation of histone H3 (panel E) and expression of HER2 and histone H3 (panels F and G respectively) in response to doxorubicin (doxo; 100 nM) and/or the CUCD-101 inhibitor (CUDC; 1 µM). Heat-shock protein 90 (HSP90, panel H) was used as loading control.


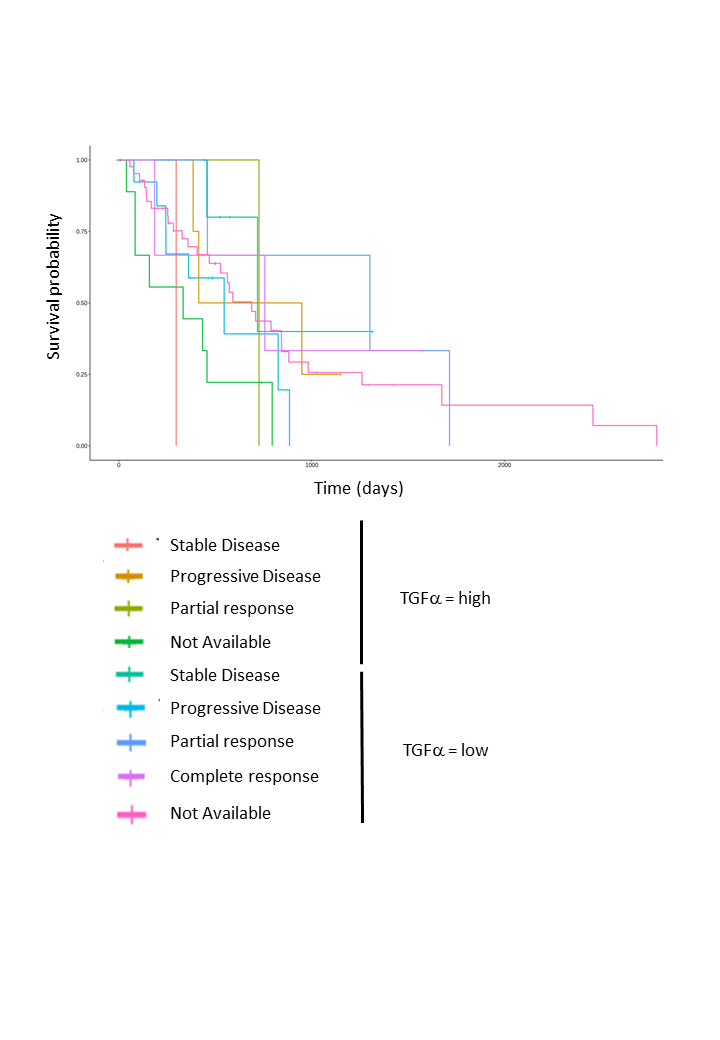


**Figure S8.** Stratification of survival rate based on response to treatment and TGFα expression. A Kaplan-Meier survival analysis integrating gene expression (low or high) and drug response (stable disease versus partial response) was performed using the TCGA dataset.

**Table S2.** Levels of VPA-doxorubicin-induced apoptosis and TGF-α expression in 10 MPM cell lines.

| Cell Line  Name | Histologic Subtype | Cellosaurus  CVCL Number | Apoptosis Induced by VPA+Doxorubicin (%) | | Log10[normalized TGF-α mRNA Level] | |
| --- | --- | --- | --- | --- | --- | --- |
|  |  |  | *Mean* | *SD* | *Mean* | *SD* |
| H2452 | biphasic | CVCL_1553 | 4.89 | 2.29 | 1.30 | 0.11 |
| M38K | biphasic | CVCL_8108 | 14.58 | 2.61 | 1.27 | 0.05 |
| MSTO-211H | biphasic | CVCL_1430 | 35.66 | 1.14 | 0.48 | 0.26 |
| SPC111 | biphasic | CVCL_D311 | 10.87 | 6.31 | 1.64 | 0.12 |
| SPC212 | biphasic | CVCL_D312 | 4.15 | 1.23 | 0.93 | 0.17 |
| ZL5 | biphasic | CVCL_5907 | 9.17 | 3.76 | 2.29 | 0.18 |
| M14K | epitheloid | CVCL_8102 | 20.53 | 3.90 | 1.46 | 0.10 |
| ZL55 | epitheloid | CVCL_5908 | 6.43 | 2.75 | 1.24 | 0.27 |
| H28 | sarcomatoid | CVCL_1555 | 4.00 | 1.66 | 3.11 | 0.10 |
| ZL34 | sarcomatoid | CVCL_5906 | 2.74 | 1.96 | 3.23 | 0.21 |

Apoptosis was measured by quantifying the proportion of sub-G1 cells by flow cytometry. TGFα mRNA levels were quantified by RT-qPCR and normalized to HPRT. SD = standard deviation.
